# Relationship of *PIK3CA* mutation and pathway activity with antiproliferative response to aromatase inhibition

**DOI:** 10.1186/bcr3683

**Published:** 2014-06-30

**Authors:** Elena López-Knowles, Corrinne V Segal, Qiong Gao, Isaac Garcia-Murillas, Nicholas C Turner, Ian Smith, Lesley-Ann Martin, Mitch Dowsett

**Affiliations:** 1 Academic Department of Biochemistry, Royal Marsden Hospital, Fulham Road, London SW3 6JJ, UK; 2 Breakthrough Breast Cancer Research Centre, Institute of Cancer Research, Fulham Road, London SW3 6JB, UK

## Abstract

**Introduction:**

*PIK3CA* (phosphatidylinositol-4,5-bisphosphate 3-kinase, catalytic subunit α) somatic mutations are the most common genetic alteration in breast cancer (BC). Their prognostic value and that of the phosphatidylinositol 3-kinase (PI3K) pathway in BC remains only partly defined. The effect of *PIK3CA* mutations and alterations of the PI3K pathway on the antiproliferative response to aromatase inhibitor treatment was determined.

**Methods:**

The Sequenom MassARRAY System was used to determine the presence of 20 somatic mutations across the *PIK3CA* gene in 85 oestrogen receptor–positive (ER+) BC patients treated with 2 weeks of anastrozole before surgery. Whole-genome expression profiles were used to interrogate gene signatures (GSs) associated with the PI3K pathway. Antiproliferative activity was assessed by the change in Ki67 staining between baseline and surgery. Three GSs representing the PI3K pathway were assessed (*PIK3CA*-GS (Loi), *PI3K*-GS (Creighton) and *PTEN*-loss-GS (Saal)).

**Results:**

In our study sample, 29% of tumours presented with either a hotspot (HS, 71%) or a nonhotspot (non-HS, 29%) *PIK3CA* mutation. Mutations were associated with markers of good prognosis such as progesterone receptor positivity (PgR+) (*P* = 0.006), low grade (*P* = 0.028) and luminal A subtype (*P* = 0.039), with a trend towards significance with degree of ER positivity (*P* = 0.051) and low levels of Ki67 (*P* = 0.051). Non-HS mutations were associated with higher PgR (*P* = 0.014) and ER (*P* < 0.001) expression than both wild-type (WT) and HS-mutated samples, whereas neither biomarker differed significantly between WT and HS mutations or between HS and non-HS mutations. An inverse correlation was found between the Loi signature and both the Creighton and Saal signatures, and a positive correlation was found between the latter signatures. Lower pretreatment Ki67 levels were observed in mutation compared with WT samples (*P* = 0.051), which was confirmed in an independent data set. Mutation status did not predict change in Ki67 in response to 2 weeks of anastrozole treatment; there was no significant difference between HS and non-HS mutations in this regard.

**Conclusions:**

*PIK3CA* mutations are associated with classical markers of good prognosis and signatures of PI3K pathway activity. The presence of a *PIK3CA* mutation does not preclude a response to neoadjuvant anastrozole treatment.

## Introduction

About 80% of primary breast cancer (BC) presents as oestrogen receptor–positive (ER+) disease [1]. In postmenopausal patients, aromatase inhibition is the most effective means of treatment, reducing the relative risk of recurrence by 20% to 25% from the results achieved with tamoxifen therapy. However, resistance remains a substantial problem and is the focus for the development of new therapeutic strategies targeting putative resistance mechanisms.

To date, although researchers in *in vitro* studies have reported large numbers of putative mechanisms of resistance, few of these mechanisms have been confirmed as clinically relevant (for example, loss of ER expression, *de novo* or acquired overexpression of human epidermal growth factor receptor 2 (HER2)) [2]. Detailed molecular characterisation of individual tumours, along with assessment of clinical efficacy, is required in order to identify additional mechanisms and determine their importance. This is particularly relevant, given that multiple new agents with targets that have putative involvement in endocrine resistance are now available [3].

The phosphatidylinositol 3-kinase (PI3K) pathway is frequently altered in cancer and is a focus of interest in ER + BC. This pathway links the signalling of type I and type II receptor tyrosine kinases (epidermal growth factor (EGFR), HER2 and insulin-like growth factor 1 receptor) to the effector serine/threonine kinase moiety AKT. Complete phosphorylation of AKT results in activation of the mammalian target of rapamycin (mTOR)/regulated associated protein of TOR (Raptor) complex 1 (mTORC1), which regulates cell proliferation and protein synthesis [3]. The phosphatase and tensin homolog (*PTEN*) tumour suppressor gene, amongst others, is a negative regulator of the PI3K pathway and is often lost in BC [4], leading to enhanced activation of AKT and downstream partners.

Whilst there are multiple isoforms of the PI3K regulatory and catalytic subunits, single amino acid substitutions in BC are frequently found in the catalytic subunit p110α (*PIK3CA*), in which the occurrence of mutations is reported to be between 18% and 41% [4-15]. Within *PIK3CA*, there are three major hotspots (HSs) of mutations concentrated in the helical (E542K and E545K) and kinase (H1047R) domains, accounting for approximately 89% of *PIK3CA* mutations [13].

*PIK3CA* mutations have been reported to be associated with markers of good prognosis, such as high ER, high PgR, smaller size, earlier stage, lower grade [5-7,13,14] and better outcome in ER+/HER2- disease, as well as with good prognosis with tamoxifen monotherapy, relapse-free survival, overall survival and BC-specific survival [5,6,16]. In contrast, BC patients with *PIK3CA* mutations and HER2 amplification have been found to exhibit significantly shorter progression-free survival following trastuzumab-based treatment [17]. However, in some cases, study researchers who have reported conflicting results have been unable to verify these associations [10,13,16].

Recognition of the frequency of *PIK3CA* mutations in clinical specimens and their oncogenic nature *in vitro* has led to the clinical development of numerous inhibitors of the pathway. The appropriate targeting of these agents in postmenopausal patients with ER + disease requires an improved understanding of the importance of *PIK3CA* mutations and PI3K pathway activation to BC response or resistance to aromatase inhibition. This insight should have a significant impact on the design and interpretation of clinical trials of endocrine therapy with PI3K pathway–targeted inhibitors.

Study of the influence of putative mechanisms and markers of resistance in early disease is not possible at an individual patient level in relation to benefit from adjuvant therapy, owing to the absence of reliable markers of subclinical disease and the inability to characterise the molecular nature of micrometastatic disease. In contrast, presurgical treatment allows the response of primary disease (unaffected by previous therapy) to be characterised by multiple means and to be correlated with the molecular characteristics. The nuclear proliferation marker Ki67 has been validated as an intermediate endpoint of evaluation of benefit from endocrine therapy in multiple studies, and it is therefore helpful for studying both resistance and the impact of targeted therapies alongside endocrine agents [18].

We therefore conducted a comprehensive assessment of *PIK3CA* mutation status and gene signatures (GSs) of PI3K pathway activity in tumours from patients with ER + primary BC in a clinical trial of the aromatase inhibitor anastrozole and related these to the drug’s antiproliferative effects and other key biomarkers of prognosis in ER + disease.

## Methods

### Tissue specimens

All available pretreatment (baseline) core-cut tumour samples were selected for analysis from patients who received the aromatase inhibitor anastrozole alone prior to biopsy in the multicentre 0223 neoadjuvant study (ZD1839IL/0223) of anastrozole with or without EGFR inhibitor gefitinib in ER + primary BC [19]. The original study received approval from an institutional review board at each site and was conducted in accordance with the 1964 Declaration of Helsinki and the International Conference on Harmonization/Good Clinical Practice guidelines. Written informed consent was obtained from each patient before participation. Tumour size was measured by caliper, magnetic resonance imaging, physical palpation or ultrasound as per the preference of each centre. Previously reported scores for ER, PgR, HER2 (positivity (+) or negativity (-) by immunohistochemistry (IHC) and fluorescence *in situ* hybridisation) and Ki67 levels (percentage of positive cells) were available [19]. ER and PgR expression were assessed by H-score, which incorporates both the staining intensity (scored from 0 to 3) and the percentage of cells stained. For both biomarkers, we used an H-score ≥1 as a cut-off for designation of positive or negative status in accordance with the American Society of Clinical Oncology/College of American Pathologists guidelines for ER and PgR positivity [20].

Percent residual Ki67 was defined as (2-week Ki67 level ÷ baseline Ki67 level) × 100. This percentage was used as an endpoint for treatment response, both as a continuous variable and dichotomised into responders or nonresponders according to a 2-week/pre-Ki67 residual <65% or > 65%, respectively [19]. This cut-off was a prospectively defined primary endpoint of the original study and has previously been shown to predict clinical response to hormone therapy.

### Validation data set

Tumours from a set of 310 pretreatment ER + patients, predominantly HER2-, from 2 neoadjuvant aromatase inhibitor studies were used for confirmation of the relationship of Ki67 data with *PIK3CA* mutation status [9].

### Gene expression arrays

Whole-genome expression data for 66 pretreatment biopsies and their derivation have been described in detail previously [21]. Intrinsic subtyping was assigned using the 50-gene predictor for luminal A and luminal B subtypes together with the gene expression data [22].

### *PIK3CA* mutation analysis

Multiple (one to three) 10-μm sections were taken from formalin-fixed, paraffin-embedded material, stained with nuclear fast red and needle-dissected for tumour-enriched areas using adjacent haematoxylin and eosin–stained sections as a guide, with the aim of yielding >70% malignant tissue. DNA was extracted using the DNeasy Tissue & Blood Kit (Qiagen, Hilden, Germany) with xylene and ethanol washes to remove paraffin and an overnight incubation in 1 M NaSCN. Nonsynonymous somatic *PIK3CA* mutations previously described in BC patients were selected from public databases, including COSMIC [23] and the National Cancer Institute’s somatic mutation database [24]. Mutation detection was conducted by using the Sequenom MassARRAY System (Sequenom, San Diego, CA, USA) as described by others [25]. In brief, with this array, DNA is amplified and treated with shrimp alkaline phosphatase to neutralise unincorporated deoxyribonucleotide triphosphates, the mutation site is extended and the sample is read by matrix-assisted laser desorption/ionisation time-of-flight (MALDI-TOF) mass spectrometry analysis. PCR and extension primers were designed using Sequenom Assay Design Software (Sequenom) and covered 20 mutations (3 HS and 17 low-frequency mutations) using 5 multiplexes (Additional file 1: Table S1). To be considered mutated, the proportion of the mutant allele had to be ≥10% as calculated by the allelotyping function of the Sequenom system.

### Droplet digital PCR

HS mutations identified by mass spectrometry–based genotyping were confirmed using a droplet digital PCR (ddPCR) system (Bio-Rad Laboratories, Hercules, CA, USA). Digital PCRs were performed using this system with *PIK3CA* primers and probes (described in Additional file 2: Table S2) at final concentrations of 900 nM for primers and 250 nM for probes.

PCRs were prepared with 10 μl of master mix in a total volume of 20 μl and partitioned into approximately 14,000 droplets per sample in the droplet generator component of the QX100 ddPCR system according to the manufacturer’s instructions. Emulsified PCRs were run on a 96-well plate on a G-Storm GS4 thermal cycler (G-Storm, Somerton, UK). The plates were incubated at 95°C for 10 minutes, followed by 45 cycles at 95°C for 15 seconds and 63.1°C for 60 seconds (exon 9), or 45 cycles at 95°C for 15 seconds and 61.7°C for 60 seconds (exon 20), and then by a 10-minute incubation at 98°C. The temperature ramp increment was 2.5°C/s for all steps. Plates were read on a QX100 droplet reader with QuantaSoft v1.3.2.0 software (both from Bio-Rad Laboratories) to assess the number of droplets positive for *PIK3CA* mutant (*PIK3CA*mu), wild type (WT), both, or neither. At least two negative control wells with no DNA were included in every run.

The concentration of *PIK3CA* mutant DNA (copies of *PIK3CA*mu DNA per droplet) was estimated based on the Poisson distribution using the number of *PIK3CA*mu copies per droplet, M_PIK3CAmu_ = -ln(1 - (*n*_PIK3CAmu_/*N*)), where *n*_PIK3CAmu_ is the number of droplets positive for the *PIK3CA* 6-carboxyfluorescein (FAM) probe and *N* is the total number of droplets. The DNA concentration in the reaction was estimated as follows: M_DNAconc_ = -ln(1 - (*n*_DNAconc_/*N*)), where *n*_DNAconc_ is the number of droplets positive for *PIK3CA* FAM probe and *PIK3CA* VIC probe (Life Technologies, Carlsbad, CA, USA) and *N* is the total number of droplets. The fraction mutation is M_PIK3CAmu_/M_DNAconc_.

### Gene signatures

Multiple GSs have been developed to represent the PI3K pathway, such as the *PIK3CA*-GS [6], *PTEN*-loss-GS [26] and *PI3K-*GS [27]. We reproduced each signature by identifying the genes that were represented on the array (Illumina, Inc, San Diego, CA, USA) and, where multiple probes existed, taking the most variable of those probes. The GS score for each sample was calculated by performing gene centring and then weighted averaging as previously described [6]:

$$\mathrm{Gene}\;\mathrm{signature}\;\mathrm{score}\left(s\right)=\sum_{i=1}^{n}W_{i}X_{i}/\sum \left|W_{i}\right|,$$

where *n* is the number of genes in a module, *X*_i_ represents the normalised gene expression in the sample and gene-specific weights *W*_i_ are equal to +1 or -1 according to the direction of their association with the phenotype in the original publication.

*PIK3CA-*GS (Loi), *PTEN*-loss-GS (Saal) and *PI3K*-GS (Creighton) were also adapted by the removal of proliferation-associated genes (PAGs) to generate three new signatures (*PIK3CA*-noPAG-GS, *PTEN*-loss-noPAG-GS and *PI3K*-noPAG-GS), because the presence of multiple PAGs dominate the GSs and could potentially mask the PI3K pathway–related features of the GSs. These PAGs were identified by three methods: genes functionally associated with cell-cycle progression and cell division according to Gene Ontology annotations [28], genes showing cell-cycle stage–specific expression [28,29] and a tumour-based ‘proliferation cluster’ [28,30-32]. The details of the genes used from each GS are listed in Additional file 3: Table S3.

### Statistical analyses

All statistical analyses were performed in GraphPad Prism 6.0 (GraphPad Software, La Jolla, CA, USA) and R version 3.0.1 (R Foundation for Statistical Computing [http://www.r-project.org/]) or in MS Excel (Microsoft, Redmond, WA, USA). Associations between tumour size (T2 or T3), nodal status (positive or negative for nodal involvement), stage (2, 3 or 4), grade (1, 2 or 3), histology (ductal, lobular or other), HER2 positivity, intrinsic subtypes (luminal A vs luminal B) and *PIK3CA* mutation status were assessed using Fisher’s exact test. The association between continuous variables, such as ER, PgR, Ki67 and GS scores, with mutation status was evaluated using the nonparametric Mann–Whitney *U* test or Kruskal–Wallis test with Dunnett’s multiple comparison test. For HS vs non-HS comparisons, if a sample contained both types of mutation, it was excluded from the analysis. Correlations between the GS scores and Ki67 IHC values were calculated using Spearman’s rank correlation analysis. All *P*-values reported are two-sided, and values <0.05 were considered statistically significant. Cases with missing values were excluded. The number of samples within each analysis can be found in the relevant analysis and in Additional file 4: Table S4.

## Results

### *PIK3CA* mutations in ER + breast cancer

DNA from tumours of 126 patients included in the 0223 trial was available, among which 19 samples were not assessed because of a lack of sufficient material. We processed 107 samples using the Sequenom system, which provided us with complete mutational profiles for 85 samples to which the data reported here relate. The demographics of the 85 patients are shown in Table 1.

**Table 1:** **Relationship between *PIK3CA *** **mutation status and standard clinicopathological parameters**^**a**^

	**Number of patients (% in brackets)**			
**Demographic characteristics**	**Total population number (%)**	***PIK3CA *** **WT, *n *** **(%)**	***PIK3CA *** **mutation, *n *** **(%)**	***P*-value**^**b**^
Totals	85 (100)	60 (70.6)	25 (29.4)	
Mean age (±SD), yr	70.9 (8.5)	71.0 (8.4)	70.7 (9.0)	
Histological grade				0.033
Number available	70	47	23	
1	13 (18.6)	5 (10.6)	8 (34.8)	
2	43 (61.4)	30 (63.8)	13 (56.5)	
3	14 (20.0)	12 (25.5)	2 (8.7)	
Unknown	15			
Stage (*n* = 85)				0.780
2	48 (56.5)	33 (55.0)	15 (60.0)	
3	28 (32.9)	21 (35.0)	7 (28.0)	
4	9 (10.6)	6 (10.0)	3 (12.0)	
Lymph nodes (*n* = 85)				0.472
Positive	35 (41.2)	23 (38.3)	12 (48.0)	
Negative	50 (58.8)	37 (61.7)	13 (52.0)	
Tumour size^c^ (*n* = 76)				0.610
T2	48 (63.2)	33 (61.1)	15 (68.2)	
T3	28 (36.8)	21 (38.9)	7 (31.2)	
Histology				0.725
Number available	58	38	20	
Ductal	44 (75.9)	29 (76.3)	15 (75)	
Lobular	8 (13.8)	6 (15.8)	2 (10)	
Other	6 (10.3)	3 (7.9)	3 (15)	
Unknown	27			
ERα status				1.000
Number available	83	25	58	
Negative	0	0	0	
Positive	83 (100)	25 (100)	58 (100)	
Unknown	2			
PgR status				0.49
Number available	83	58	25	
Negative	11 (12.9)	9 (15.5)	2 (8)	
Positive	72 (86.7)	49 (84.5)	23 (92)	
Unknown	2			
HER2 status				1.000
Number available	82	57	25	
Negative	69 (84.1)	48 (84.2)	21 (84)	
Positive	13 (15.9)	9 (15.8)	4 (16)	
Unknown	3			
Subtype				0.039
Number available	46	23	13	
Luminal A	22 (47.8)	11 (47.8)	11 (84.6)	
Luminal B	24 (52.2)	12 (52.2)	2 (15.4)	

^a^ER, Oestrogen receptor; HER2, Human epidermal growth factor receptor 2; PgR, Progesterone receptor; *PIK3CA*, Phosphatidylinositol-4,5-bisphosphate 3-kinase, catalytic subunit α; WT, Wild type. ^b^Fisher’s exact test. *P*-value relates to the comparison between wild type and mutation. ^c^Measurements of tumour size were taken by caliper (*n* = 45), magnetic resonance imaging (*n* = 2), physical examination (*n* = 25) or ultrasound (*n* = 4). T4 and T4a patients (*n* = 1 and *n* = 8, respectively) were excluded from the analysis of tumour size.

**Figure 1:**
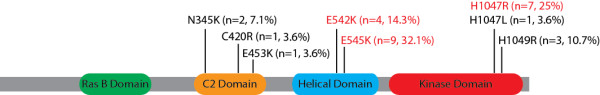
**Representation of the frequency of mutations identified in the *PIK3CA *gene.** Mutations in the hotspot regions of the gene are coloured red. The percentage for each type is the percentage of total mutations recorded.

At least one *PIK3CA* mutation was detected in 25 tumours (29%). Of those 25, 23 had one mutation, 1 had 3 mutations (exon 7 E453K, exon 9 E542K and E545K) and another had 2 mutations (exon 9 E545K and exon 20 H1047R). Of the 28 mutations, 20 (71%) were in HSs: 14% E542K (*n* = 4), 32% E545K (*n* = 9) and 25% H1047R (*n* = 7) (Figure 1, Additional file 5: Table S5).

The presence of E542K, E545K and H1047L/R mutations was also assessed by ddPCR in 25 samples with mutations, including 19 samples that contained 1 of these mutations, which were revealed by Sequenom analysis. Nineteen of the twenty-one mutations were confirmed by ddPCR. Of the two that were not confirmed, one was below the threshold for sample calling (>25 VIC + droplets) by ddPCR because of low quantity of DNA and the other was discordant. However, as a result of this excellent correlation, the mutations detected with the Sequenom system were carried forward for downstream analyses.

### Relationship of *PIK3CA* mutations with clinicopathological features

**Figure 2:**
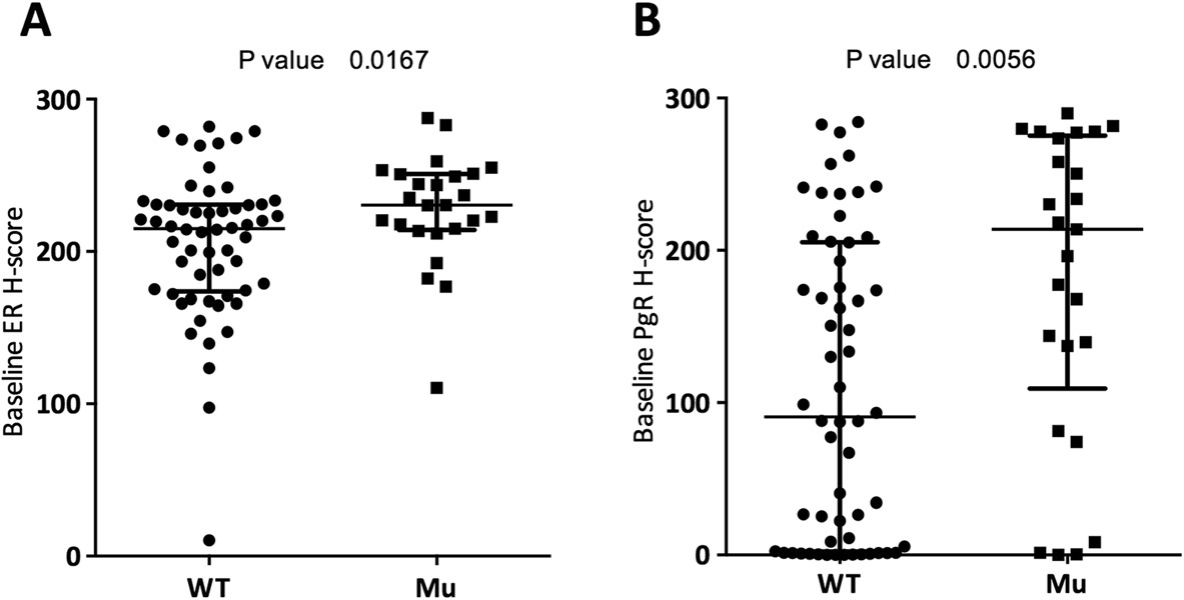
**Relationship of *PIK3CA *mutation status with biomarkers and molecular subtype. (A)** Oestrogen receptor (ER) (*n* = 83). **(B)** Progesterone receptor (PgR) (*n* = 83). Data are shown with medians and IQRs. Mu, Mutant; WT, Wild type.

The presence of any mutation was associated with low-grade tumours (*P* = 0.033), but not with size as characterized by T category (T2 vs T3; *P* = 0.61) (Table 1). Quantitative ER and PgR protein expression (assessed by H-score) was available for 83 of 85 cases. In patients with *PIK3CA* mutations, the median ER levels were significantly higher: WT = 215.1 (*n* = 58, IQR = 173.7 to 230.9) and Mu = 230.5 (*n* = 25, IQR = 214.3 to 251.1) (*P* = 0.017) (Figure 2A). Amongst the mutation cases, median PgR H-scores were also significantly higher: WT = 90.6 (*n* = 58, IQR = 2.25 to 205.3) and Mu = 213.8 (*n* = 25, QR = 109.4 to 275.6) (*P* = 0.006) (Figure 2B). All except two of the mutation cases were PgR+. Despite this, when PgR was dichotomised as positive or negative, no significant association with mutation status was identified (Table 1).

ER H-score and PgR H-score were also analysed with regard to HS and non-HS mutations. We found that non-HS mutations exhibited significantly higher ER and PgR expression compared to WT and HS samples. For ER, the median expression data were as follows: WT = 215.1 (*n* = 58, IQR = 173.7 to 230.9), HS = 220.4 (*n* = 17, IQR = 202.2 to 247.7), non-HS = 249.4 (*n* = 7, IQR = 235.4 to 255.2), HS vs non-HS/WT = ns, WT vs non-HS (*P* < 0.001). For PgR expression, the median values were as follows: WT = 90.60 (*n* = 58, IQR = 2.25 to 205.3), HS = 168.0 (*n* = 17, IQR = 77.7 to 224.4) and non-HS = 258.3 (*n* = 7, IQR = 196.2 to 281.9), with a nonsignificant correlation between HS and non-HS/WT and a significant difference between WT and non-HS (*P* = 0.014). One sample contained both a HS mutation and a non-HS mutation and was therefore excluded from the analysis.

Regarding the relationship between HER2 status and *PIK3CA* mutations, 69 cases were HER2- and harboured 21 mutations and 13 cases were HER2+ and contained 4 mutations (*P* = ns) (Table 1).

Intrinsic subtype status according to the PAM50 GS [22] was available for 66 of 85 samples, among which we classified 22 as luminal A, 14 as luminal B, 17 as normal, 3 as basal-like and 10 as HER2-enriched. There were 11 (50%) of 22 luminal A and 2 (14%) of 14 luminal B mutation cases (*P* = 0.039) (Table 1). Thus, 11 (85%) of the 13 mutation cases were categorised as luminal A.

### Signatures of phosphatidylinositol 3-kinase pathway activity

Three GS scores were calculated for each sample at baseline. The *PIK3CA*-GS (Loi) was derived from exon 20 mutations and has been shown to negatively correlate with GSs of proliferation, AKT activation and PTEN loss whilst positively correlating with *ESR1* expression [6]. The *PTEN*-loss-GS (Saal) was developed to represent IHC-detectable PTEN loss in BC [26]. Its association with *PIK3CA* mutation status suggests that the signature integrates various lesions in the PI3K pathway. The *PI3K*-GS (Creighton) is based on the set of genes in which expression was induced or repressed by PI3K inhibitors [27]. As measurements of the same pathway, it might be expected that the signatures would correlate with one another. However, although the *PI3K*-GS (Creighton) and *PTEN*-loss-GS (Saal) were strongly positively correlated with one another (*r* = 0.657, *P* < 0.0001), the *PIK3CA*-GS (Loi) and *PI3K-*GS (Creighton) showed a weak, nonsignificant trend towards negative correlation (*r* = -0.218, *P* = 0.079), and the *PIK3CA-GS* (Loi) and *PTEN*-loss-GS (Saal) were minimally inversely correlated (*r* = -0.292, *P* = 0.017). The *PIK3CA*-GS (Loi) has been reported to correlate with *ESR1*[6], but we did not see this relationship (*r* = -0.122, *P* = 0.330). It did, however, show a slight trend towards correlation with ER protein expression (*r* = 0.240, *P* = 0.055).

### *PIK3CA* mutation status and gene signatures representing the phosphatidylinositol 3-kinase pathway

**Figure 3:**
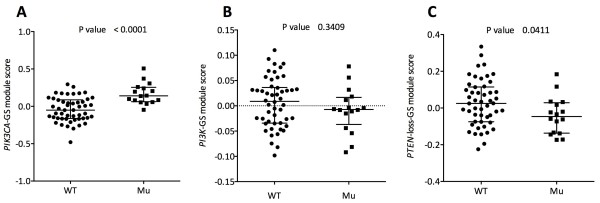
**Relationship between *PIK3CA *mutation status and genetic signatures. (A)***PIK3CA*-GS (Loi). **(B)***PI3K*-GS (Creighton). **(C)***PTEN*-loss-GS (Saal). Data are shown with medians and IQRs. GS, Gene signature; Mu, Mutant; WT, Wild type. *n* = 66.

The *PIK3CA-*GS (Loi) was strongly associated with mutation status, with mutants having higher median GS scores: WT = -0.051 (*n* = 50, IQR = -0.162 to 0.086) and Mu = 0.140 (*n* = 16, IQR = 0.062 to 0.254) (*P* < 0.0001) (Figure 3A). Because this signature was derived using exon 20 mutations, we ran the same analysis excluding the exon 20 mutations and found a significant relationship with non–exon 20 mutations: WT = -0.051 (*n* = 50, IQR = -0.162 to 0.086) and Mu = 0.143 (*n* = 11, IQR = 0.056 to 0.240) (*P* = 0.0003) (data not shown).

The *PI3K*-GS (Creighton) was not associated with *PIK3CA* mutation status. Median values were WT = 0.009 (*n* = 50, IQR = -0.034 to 0.036) and Mu = -0.007 (*n* = 16, IQR = -0.037 to 0.016) (*P* = 0.341) (Figure 3B). The *PTEN*-loss-GS (Saal) was significantly inversely associated with mutation status. Median values were WT = 0.025 (*n* = 50, IQR = -0.074 to 0.115) and Mu = -0.047 (*n* = 16, IQR = -0.137 to 0.029) (*P* = 0.041) (Figure 3C).

When we excluded PAGs from the GSs, the association with mutation status was maintained for the *PIK3CA-*GS (Loi) and the *PI3K*-GS (Creighton). For *PIK3CA-*GS (Loi), the median values were WT = -0.041 (*n* = 50, IQR = -0.166 to 0.095) and Mu = 0.151 (*n* = 16, IQR = 0.051 to 0.259) (*P* < 0.0001) (data not shown). For *PI3K*-GS (Creighton), the median values were WT = 0.006 (*n* = 50, IQR = -0.031 to 0.039) and Mu = -0.002 (*n* = 16, IQR = -0.035 to 0.021) (*P* = 0.372) (data not shown). However, the *PTEN*-loss-GS (Saal) association lost its statistical significance. The median values for *PTEN*-loss-GS (Saal) were WT = -0.002 (*n* = 50, IQR = -0.049 to 0.089) and Mu = -0.007 (*n* = 16, IQR = -0.081 to 0.041) (*P* = 0.503) (data not shown), indicating that this GS is influenced by proliferation genes (27.8% of genes in the GS are PAGs, compared to 12% for *PIK3CA*-GS and 15% for *PI3K*-GS).

### Association of *PIK3CA* mutation status and gene signatures with Ki67

There was a borderline significant difference (*P* = 0.051) in baseline Ki67 between *PIK3CA* WT and mutation samples, with patients with the mutation having lower levels of proliferation (Table 2). We also analysed baseline Ki67 with regard to HS and non-HS mutations and found no evidence for a distinction to be made (*P* = 0.144).

**Table 2:** **Relationship between Ki67 and *PIK3CA *** **mutation status**^**a**^

		**WT/Mu**		
	***n***	**WT, median (IQR)**	**Mu, median (IQR)**	***P*-value**
Baseline Ki67	81	18.2 (56, 12.7 to 27.3)	12.5 (25, 5.7 to 21.0)	0.051
2-week Ki67	75	5.8 (52, 1.8 to 19.8)	3.1 (23, 1.0 to 7.2)	0.050
2-wk/pre-Ki67%	73	31.3 (50, 14.5 to 70.8)	23.1 (23, 11.6 to 44.2)	0.346

^a^Mu, Mutant; WT, Wild type.

There was no significant correlation between baseline Ki67 and the *PIK3CA*-GS (Loi); however, there was a significant positive correlation with the *PI3K*-GS (Creighton) and *PTEN*-loss-GS (Saal) (Table 3). Because the various GSs include PAGs, the influence of these PAGs on the signatures could be considered substantial because proliferation has been strongly associated with the predictive and prognostic utility of gene modules [33]. Therefore, three modified GSs (noPAG version) which had PAGs removed were developed. In the absence of PAGs, the relationships of baseline Ki67 with the GSs were sustained.

**Table 3:** **Relationship between Ki67 and phosphatidylinositol 3-kinase pathway gene signatures**^**a**^

		**With PAGs**						**Without PAGs**					
		***PIK3CA*-GS (Loi)**		***PI3K*-GS (Creighton)**		***PTEN*-loss-GS (Saal)**		***PIK3CA*-noPAG-GS (Loi)**		***PI3K*-noPAG-GS (Creighton)**		***PTEN*-loss-noPAG-GS (Saal)**	
	***n***	**Spearman’s *r***	***P***-**value**	**Spearman’s *r***	***P***-**value**	**Spearman’s *r***	***P***-**value**	**Spearman’s *r***	***P***-**value**	**Spearman’s *r***	***P***-**value**	**Spearman’s *r***	***P***-**value**
Baseline Ki67	64	-0.101	0.427	0.371	0.003	0.508	<0.0001	-0.082	0.518	0.370	0.003	0.347	0.005
2-week Ki67	57	0.084	0.534	0.188	0.162	0.219	0.102	0.120	0.374	0.186	0.167	0.132	0.329
2-wk/pre-Ki67%	56	0.212	0.117	0.036	0.792	-0.004	0.976	0.246	0.068	0.033	0.811	-0.017	0.901

^a^GS, Gene signature; PAG, Proliferation-associated gene.

Ki67 values after 2 weeks of treatment have been found to be associated with residual risk during endocrine treatment [20]. We found a weak significant relationship of mutation patients’ having lower levels of 2-week Ki67 (*P* = 0.050) (Table 2), but no association with any of the GSs, with or without PAGs (Table 3).

Ki67 levels were suppressed by median values of 69% and 77% in patients with WT and mutation GSs, respectively, but this difference was not statistically significant (Table 2 and Additional file 6: Figure S1). Similarly, when we divided the patients into good and poor responders, using a cut-off value of 65%, we identified no difference in the proportion of good and poor responders in the WT and mutation groups (*P* = 0.095) (data not shown). In addition, there was no significant correlation between any of the three GSs and 2-week pre-Ki67% measurements (Table 3). We identified a slight trend towards significance for *PIK3CA*-noPAG-GS (*r* = 0.246, *P* = 0.067) (Table 3 and Additional file 7: Figure S2). There were ten HER2+ samples with mutation status and 2-week/pre-Ki67% data; however, the small sample size precluded any meaningful separate analysis of this subgroup.

### Validation of Ki67 findings with regard to mutation status using a publicly available data set

We conducted an analysis of 310 baseline samples from the study by Ellis *et al*. [9] for which they had *PIK3CA* mutation data. In this much larger data set, we found a significant but weak association with baseline Ki67 and discovered that WT samples had higher Ki67 levels than mutation samples, with the following median values: WT = 18.6 (*n* = 180, IQR = 10.3 to 31.4) and Mu = 14.9 (*n* = 130, IQR = 7.3 to 26.7) (*P* = 0.043). This confirms the trend we identified in our smaller cohort. Unlike our data, there was no trend towards mutation samples’ having lower 2-week Ki67 values, with median data as follows: WT = 3.3 (*n* = 66, IQR = 1.2 to 8.3) and Mu = 3.4 (*n* = 40, IQR = 1.2 to 7.8) (*P* = 0.926). In our analysis of Ellis *et al*.’s data using our methodology for Ki67 change, we also did not identify a significant association, with the following median data: WT = 76% suppression (*n* = 66, IQR = 46 to 92) and Mu = 72% (*n* = 40, IQR = 10 to 87) (*P* = 0.334) [9].

## Discussion

Both *de novo* and acquired resistance to endocrine therapy is a major clinical problem in the treatment of ER + BC. In recent years, there has been a concerted effort to develop therapeutic agents targeting kinases within the PI3K pathway. This has grown out of the knowledge that deregulation of this pathway is associated with with increased tumour growth and resistance to therapy [34]. Furthermore, in recent clinical studies, researchers have shown that the combination of everolimus, a targeted mTORC1 inhibitor, with the steroidal aromatase inhibitor exemestane provides greater benefit than exemestane alone in the third-line setting, increasing median progression-free survival from 4.1 to 10.6 months [35]. These data have led to a concerted effort to identify biomarkers of response to PI3K inhibitors in an attempt to identify those patients most likely to gain benefit from this therapy.

Surprisingly, in multiple studies of *PIK3CA* in BC, researchers have shown *PIK3CA* to be a highly mutated gene in the luminal subtype that is associated with good clinicopathological markers. Despite this finding, its relationship with response to endocrine therapy remains unclear. In our present study, we aimed to evaluate the relationship of *PIK3CA* mutations and GSs representing PI3K pathway activation with key biological features of BC and short-term aromatase inhibitor–induced suppression of Ki67, a validated intermediate endpoint for benefit from endocrine therapy [11,36]. To our knowledge, our present study is, to date, the largest single study of an aromatase inhibitor given in the neoadjuvant setting in which the relationship between response to treatment, *PIK3CA* mutations and multiple GSs of PI3K has been examined.

Our findings regarding the prevalence of *PIK3CA* mutations and the associations with lower risk markers in ER + BC confirm previously reported results [5,7,9,13,14,37], thus providing further evidence that mutations in this gene seem to play a role in the biology of luminal BC. To complement the mutation data, and for a better overview of the activation status of the PI3K pathway, we investigated a set of previously published GSs. *PIK3CA* mutation status was associated with both the *PIK3CA*-GS (Loi) and *PTEN*-loss-GS (Saal) pathways; however, in each case, there was substantial overlap in the GSs between WT and Mu populations, such that GS could not act as an accurate surrogate for mutation status.

The opposing association of the two signatures representing high pathway activation, *PI3K*-GS (Creighton) and *PTEN*-loss-GS (Saal), with mutation status requires careful consideration of the degree to which they represent pathway activation and the population in which they are derived. They are far from equivalent, apparently reflecting different aberrations driving the signatures. The *PI3K*-GS, derived *in vitro*, was based on repression or induction as a result of treatment with PI3K inhibitors, whereas the *PTEN*-loss-GS recapitulates IHC-detectable loss and could be more appropriate for use in studies of basal-like tumours, in which it is associated with high pathway activity [37]. With this in mind, it should also be noted that the GSs are indicative of relative pathway activation rather than absolute.

Although our analysis of *PIK3CA* mutations was comprehensive, the pathway consists of multiple nodes that could be subject to mutation (or to other aberrations, such as gain or loss of function), such that an alteration at one particular point might not have the same impact as another in terms of altering pathway activity.

Expression of Ki67 at baseline has been found to be associated with a good prognosis. In previous analyses of smaller cohorts, researchers have found that baseline Ki67 was not significantly different between WT and mutation samples overall or grouped by domain [12,16]. In this study, however, we found a borderline trend for lower levels of Ki67 in mutation samples overall. This novel finding is confirmed by our analysis of a larger sample cohort from a public data set [9]. The median and IQR Ki67 data in both our data set and that of Ellis *et al*. are extremely similar, suggesting that the weak significance in our data set is most likely due to the small sample size.

In two previous reports, researchers have described the effect of *PIK3CA* mutation status and response to treatment measured by Ki67 change in small patient cohorts with different treatments [12,16]. They identified no significant association of mutation with response. We also found no relationship in our larger, homogeneously treated cohort, and, by investigating Ki67% change in a larger public sample set [9], we confirmed that this result was not a false-negative due to sample size. The lack of a relationship between *PIK3CA* mutation status and 2-week/pre-Ki67% in all the data sets suggests that the presence of a *PIK3CA* mutation has limited, if any, impact on the likelihood of benefit from presurgical anastrozole treatment in patients with primary BC.

Interestingly, in a neoadjuvant trial of ER + postmenopausal patients, researchers found that *PIK3CA* exon 9 mutations, compared with exon 20 mutations, were less responsive to letrozole alone, but that these patients were as sensitive as the overall population to everolimus and letrozole [38]. However, the number of patients in that study was small. A pooled analysis of all published studies would allow greater exploration of the significance of the mutational subgroups because of increased power. The researchers in the recently reported BOLERO-2 and TAMRAD translational studies of ER + metastatic BC patients [39,40] found that all patients derived benefit from the addition of everolimus to exemestane or tamoxifen, respectively, regardless of their *PIK3CA* genotype. However, the relevance of *PIK3CA* mutation to advanced, heavily pretreated disease may differ from primary disease. The early emergence of *PIK3CA* mutations in BC, as well as their high prevalence and their associations with high ER expression and good prognosis, suggests their functional importance in the disease, but it may be that other aberrations offer predictive potential. For example, patients with independent activation of mTOR via low levels of the tumour suppressor LKB1, an mTOR inhibitor, exhibited greater benefit from everolimus [39].

The strengths of this study are the comprehensive documentation of the presence of both common and rare *PIK3CA* mutations (>94% of all *PIK3CA* mutations recorded in BC) and the use of multiple GSs of the PI3K pathway to catalogue and compare aspects of the PI3K pathway. The use of the presurgical setting allowed the mutations to be linked to response to treatment by using a validated biomarker, in contrast to most other studies in which researchers have considered association with prognosis. We also verified the most important novel data, lower baseline Ki67 in mutation samples, in a large, publicly available data set. The moderate sample size of this study does mean, however, that the ability to determine the importance of the rare mutations is underpowered. The numerous borderline trends could come into significance in a larger data set, as shown by the analysis of the data from Ellis and colleagues.

## Conclusions

The results of this presurgical study of 85 breast tumours treated with 2 weeks of anastrozole confirm the high prevalence of *PIK3CA* mutations and their relationship with markers of good clinical prognosis in ER + disease. The correlation of mutations with GSs of the PI3K pathway suggests that *PIK3CA* mutants exhibit lower mTORC1 signalling and reduced PI3K activity, despite their oncogenic nature *in vitro*. The data reported herein show that the presence of a *PIK3CA* mutation does not seem to attenuate the response to short-term treatment with anastrozole and therefore is not a biomarker of response as measured by Ki67, which is an important finding, given the high frequency of *PIK3CA* mutations and the widespread use of anastrozole treatment in postmenopausal women.

## Abbreviations

BC: Breast cancer; EGFR: Epidermal growth factor receptor; ER: Oestrogen receptor; FISH: Fluorescence *in situ* hybridisation; GS: Gene signature; HER: Human epidermal growth factor receptor; HS: Hotspot; IHC: Immunohistochemistry; MALDI-TOF: Matrix-assisted laser/desorption ionisation time of flight; Mu: Mutant; PAG: Proliferation-associated gene; PgR: Progesterone receptor; PI3K: Phosphatidylinositol 3-kinase; *PIK3CA*: Phosphatidylinositol-4,5-bisphosphate 3-kinase, catalytic subunit α; *PTEN*: Phosphatase and tensin homolog; WT: Wild type.

## Competing interests

The authors declare that they have no competing interests.

## Authors’ contributions

ELK extracted samples, ran the Sequenom analysis, analysed the data and wrote the manuscript. CS analysed the data and wrote the manuscript. QG provided the gene signatures. IGM and NT ran the ddPCR analysis and interpreted the data. LAM and MD conceived the study, participated in its design and coordination, and helped to draft the manuscript. IS designed the clinical study for 0223. All authors read and approved the final manuscript.

## Supplementary Material

Additional file 1: Table S1 Sequenom primer sequences. Click here for file

Additional file 2: Table S2 Primers and probes for droplet digital PCR. Click here for file

Additional file 3: Table S3 Genes comprising the signatures of Creighton *et al.*, Loi *et al*. and Saal *et al*. The coefficient describes whether the gene contributes positively or negatively to the gene signature (GS) score. “X” denotes which GS the gene was used in. The red font indicates that the gene was not mapped to the Illumina array data. The total counts for the genes that were mapped from the original publication to the Illumina array data are as follows: 176 of 181 for *PTEN*-loss-GS (Saal), 231 of 236 for *PIK3CA*-GS (Loi) and 1,733 of 1,793 for *PI3K*-GS (Creighton). Click here for file

Additional file 4: Table S4 Sample matrix. Click here for file

Additional file 5: Table S5 *PIK3CA* mutation data for the 85 patients. Click here for file

Additional file 6: Figure S1 2 wk/pre-Ki67% in wild-type (WT) and mutation (Mu) samples. Data are medians with IQRs (*n* = 73). Click here for file

Additional file 7: Figure S2 2 wk/pre-Ki67% in PI3K GS. **(A)***PIK3CA*-GS (Loi). **(B)***PI3K*-GS (Creighton). **(C)***PTEN*-loss-GS (Saal). Click here for file
