# Supplementary figures and images for: Relationship of PIK3CA mutation and pathway activity with antiproliferative response to aromatase inhibition
Source: Breast Cancer Res. 2014 Jun 30;16(3):R68. doi: 10.1186/bcr3683 (PMC4227109; doi:10.1186/bcr3683)

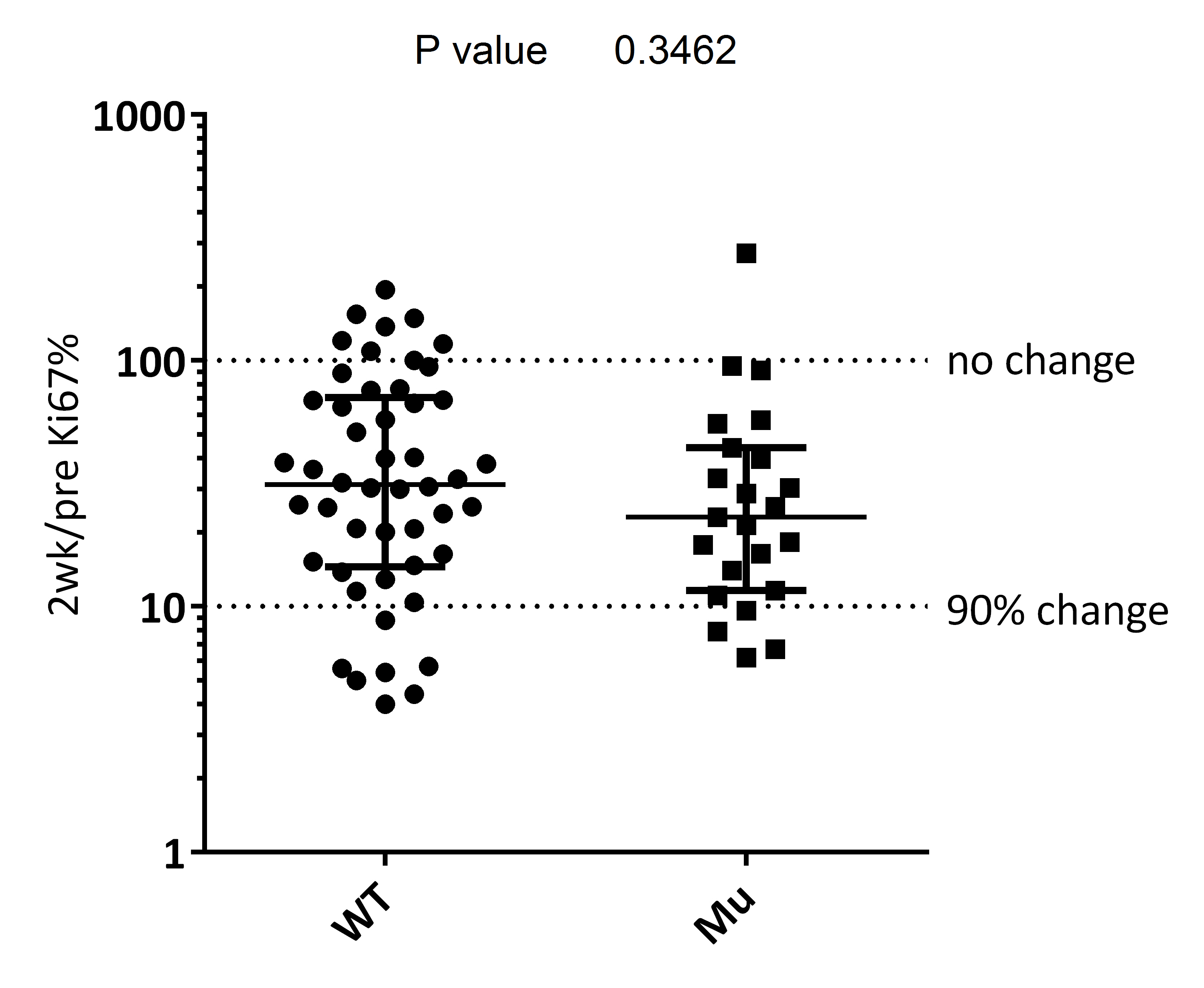

Supplement: Additional file 6: Figure S1 — 2 wk/pre-Ki67% in wild-type (WT) and mutation (Mu) samples. Data are medians with IQRs (n = 73). [file bcr3683-S6.jpeg]

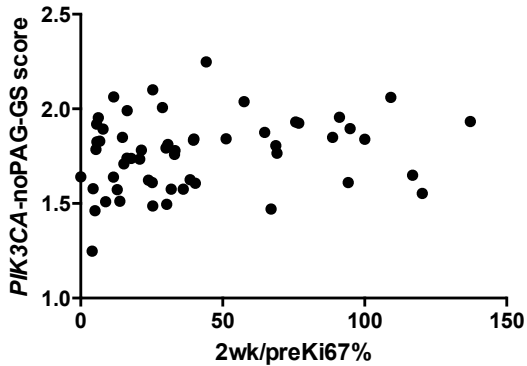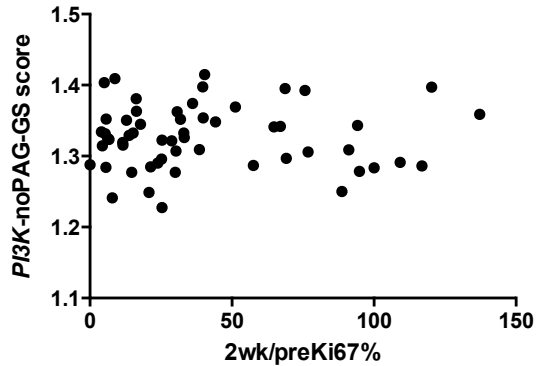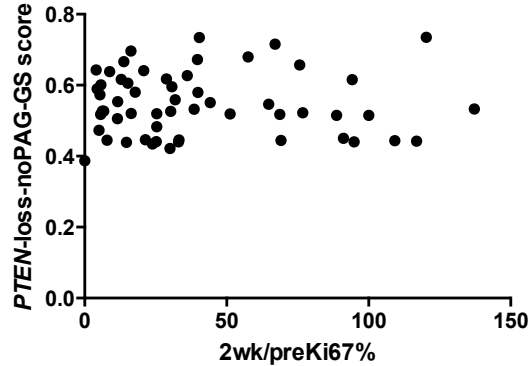

Supplement: Additional file 7: Figure S2 — 2 wk/pre-Ki67% in PI3K GS. (A)PIK3CA-GS (Loi). (B)PI3K-GS (Creighton). (C)PTEN-loss-GS (Saal). [file bcr3683-S7.pdf]
